# Correlates of susceptibility to smoking among Mexican origin youth residing in Houston, Texas: A cross-sectional analysis

**DOI:** 10.1186/1471-2458-8-337

**Published:** 2008-09-26

**Authors:** Anna V Wilkinson, Andrew J Waters, Vandita Vasudevan, Melissa L Bondy, Alexander V Prokhorov, Margaret R Spitz

**Affiliations:** 1 Department of Epidemiology, Unit 1340, The University of Texas M. D. Anderson Cancer Center, P.O. Box 301439, Houston, TX 77230-1439, USA; 2 Department of Medical and Clinical Psychology, Uniformed Services University of the Health Sciences, 4301 Jones Bridge Road Bethesda, MD 20814, USA; 3 Department of Behavioral Science, The University of Texas M. D. Anderson Cancer Center, Houston, Texas, USA

## Abstract

**Background:**

Survey data suggest that in Texas Latino youth exhibit higher rates of susceptibility to smoking than youth from other ethnic groups. In this analysis we examined the relationship between susceptibility to smoking and well-known risk factors associated with smoking initiation among a cohort of 11 to 13 year old Mexican origin youth residing in Houston, Texas.

**Methods:**

We analyzed cross-sectional survey data from 1,187 participants who reported they had never smoked, even a puff of a cigarette. The survey assessed peer and family social influence, school and neighborhood characteristics, level of family acculturation and socioeconomic status, and attitudes toward smoking. Bivariate associations, Student's t-tests, and logistic regression analysis were used to examine predictors of susceptibility.

**Results:**

Overall, 22.1% of the never-smokers were susceptible to smoking. Boys were more likely to be susceptible than girls (25.6% vs. 18.9%), and susceptible children were slightly older than non-susceptible children (12.1 vs. 11.8 years). In addition, multivariate analyses revealed that positive expectations about smoking exerted the strongest influence on susceptibility status (odds ratio = 4.85). Multivariate analyses further revealed that compared to non-susceptible participants, susceptibles were more likely to report peer influences supportive of smoking, lower subjective social status and more detentions at school, more temptations to try smoking and to have a mother and a brother who smokes.

**Conclusion:**

Our findings suggest that interventions that target positive expectations about smoking may be useful in this population. Furthermore, because youth encounter smoking-initiation risk factors in different social environments, our results underscore the continued need for both family- and school-based primary prevention programs to adequately combat their influence. The results also can be used to inform the development of culturally sensitive programs for Mexican origin youth.

## Background

The construct of cognitive susceptibility to smoking, defined as lacking a firm commitment not to smoke in the future or if offered a cigarette by a friend, integrates behavioral intentions and expectations for future behavior [1]. Over the past decade, the construct has consistently demonstrated strong predictive abilities and has become widely accepted. In prospective studies, conducted in various geographic areas of the US among adolescents from differing ethnic backgrounds, adolescents identified as susceptible to smoking were two to three times more likely to have experimented with cigarettes than their non-susceptible peers at follow-up [2-5]. Moreover, results from a community-based tobacco prevention study indicated that susceptibility to smoking is amenable to interventions [6]. In this Texas-based study, an intervention reduced susceptibility to smoking among teens by 24.6%. Collectively, these studies suggest that identifying and characterizing susceptible adolescents who have never smoked will be critical to optimizing primary smoking prevention efforts among youth.

Mexican origin youth, aged 12 to 17 years, report higher rates of susceptibility to smoking (28.8%) compared to their non-Hispanic white (20.8%) and non-Hispanic black (23.0%) counterparts [7]. Moreover, results from two large population-based surveys of middle and high school students lend support to the predictive validity of the susceptibility construct [8,9]. In Texas, Latino youth exhibit the highest rates of experimentation with cigarettes [8] and of current smoking [9], among all ethnic groups. In 2001 in Houston, Latino middle and high school students had the highest rate of experimenting with cigarettes (68.2%; 95% confidence interval [CI]: 64.2–72.2%) followed by whites (63.8%; 95% CI: 52.3–75.3%) and African Americans (53.4%; 95% CI: 48.4–58.4%). In addition, statewide in 2001, 14.5% (95% CI: 12.3–16.7%) of all Latino middle school students reported currently smoking compared to only 8.2% (95% CI: 6.3–10.2%) of white and 6.5% (95% CI: 4.5–8.4%) of African Americans middle school students. By high school, smoking rates among Latinos and whites were similar and significantly higher than smoking rates among African American and Asian students. Given that individuals of Mexican descent represent the most rapidly growing segment of the United States (US) population and account for almost 60% of the Hispanic population in the US [10], a close examination of risk factors associated with susceptibility to smoking among Mexican origin youth is timely and warranted.

Our goal in this analysis was to examine the associations between susceptibility to smoking and well-established sociodemographic, social, contextual, and behavioral risk factors associated with smoking initiation among a cohort of 11 to 13 year old Mexican origin youth in Houston, Texas. A better understanding of the relationship between susceptibility and these risk factors may facilitate the development of more effective primary smoking prevention programs targeted to this rapidly growing, understudied, and underserved population.

## Methods

### Participant recruitment

Participants included in this study were between 11 and 13 years of age and were drawn from a population-based infrastructure of Mexican American households created by the Department of Epidemiology at The University of Texas M. D. Anderson Cancer Center beginning in July 2001. Participants are self-identified Mexican Americans or Mexicans of any age and sex who reside in predominantly (at least 80% according to 2000 census) Mexican American neighborhoods in Houston. Families were recruited into the cohort through random-digit telephone dialing, block-walking, intercept (such as at health fairs), or networking through already enrolled participants. A detailed description of the recruitment methodology has been published [11].

Households with potential age-eligible participants were identified from the cohort database. The potential participants' parents or legal guardians were called to assess interest in the study. In households in which there were two or more age-eligible children, the child who had had his or her birthday most recently was invited to participate in the study. Home interviews were scheduled with all parents/legal guardians who agreed to participate. A pair of bilingual interviewers visited the home, explained the goals and scope of the study, obtained written informed consent from the child's parent/legal guardian and informed assent from the child, and enrolled the child. A total of 3,000 households were identified as eligible from the cohort database. Of these 1,425 were contacted. Just over 90% of all parents with age-eligible children who were contacted agreed to enroll their child in the study. The institutional review board at The University of Texas M. D. Anderson Cancer Center approved all aspects of this study.

### Data collection and variables available for analysis from participants

After consenting to join the study, each participant completed a 5-minute personal interview during which basic demographic (gender, age, nativity status (U.S. or Mexico)) and acculturation data were collected. Acculturation was assessed using 4 items from Marin *et al.'s *[12] Language Use subscale. The questions ascertain language used when reading, speaking at home, speaking with friends, and thinking. The scale has excellent internal reliability among Mexican Americans (alpha = 0.92). At the end of the interview, the child's height and weight were taken. The participant was then handed a personal digital assistant (PDA) with which to complete the remainder of the survey.

All measures and constructs assessed on the PDA are described in detail in Table 1. Briefly, the primary outcome variable in this study is susceptibility to smoking, which is defined as lacking a firm commitment not to smoke in the future or if offered a cigarette by a friend, integrates behavioral intentions and expectations for future behavior [1]. The construct is assessed among never-smokers only. To be coded as "non-susceptible" participants responded "no" to "Do you think you will try a cigarette soon?" and "definitely not" to "If one of your best friends were to offer you a cigarette would you smoke it?" and "Do you think you will be smoking cigarettes 1 year from now?" Risk factors associated with smoking initiation were assessed in four broad areas: attitudes towards cigarettes, peer and family influences, school characteristics and neighborhood characteristics.

**Table 1:** Measures assessed on the PDA

**Measure/Construct**	**Items**
Cognitive susceptibility to smoking [1]	3 items assessed behavioral intentions and peer influence (administered to never-smokers only). To be coded as "non-susceptible" participants responded "no" to "Do you think you will try a cigarette soon?"; and "definitely not" to "If one of your best friends were to offer you a cigarette would you smoke it?" & "Do you think you will be smoking cigarettes 1 year from now?"
***Attitudes toward smoking***	
Pros and cons of smoking [58,59]	A 12-item measure of the pros and cons of smoking (Decisional Balance Scale). Six items assessed pros of smoking, e.g., "Kids who smoke have more friends" (Cronbach's alpha = 0.72). Six items assessed cons of smoking, e.g., "Smoking is bad for people's health" (Cronbach's alpha = 0.78). Responses were made on a five-point scale ranging from "strongly disagree" to "strongly agree."
Temptations to try smoking [59]	Assessed 14 different situational temptations to try smoking, e.g. "With friends at a party", analyzed as one scale (Cronbach's alpha = 0.90). Responses were made on a five-point scale ranging from "not at all tempted" to "extremely tempted."
Positive and negative outcome expectations [17]	Seven items assessed positive expectations, e.g. "I think smoking would make me look more mature." (Cronbach's alpha = 0.86) and five items assessed negative expectations, e.g., "I think smoking would give me bad breath" (Cronbach's alpha = 0.67). Responses were made on a four-point scale ranging from "strongly disagree" to "strongly agree."
***Peer & family influence***	
Friends smoking behavior [60]	"How many of your three closest friends smoke?" (0, 1, 2, or 3) & "How many of your friends smoke?" Response options include "none," "a few," "some," "most," or "all."
Householders smoking behavior	Assessed which householders the participant currently lives with are current smokers, e.g., "Does your father smoke?"
Peer and family normative influence [61]	Assessed normative beliefs of family and peers, i.e., "How would your parents feel about your smoking cigarettes?" & "How would your close friends feel about your smoking cigarettes?" Responses were made on a four-point scale ranging from "strongly approve" to "strongly disapprove."
***School characteristics***	
Acceptability	"Do students smoke on your school campus?", "Do your friends think it is cool to smoke?", & "Do your friends think it is stupid to smoke?"
Punishment	"What happens to students who smoke at school if they get caught?" Response options include "nothing," "disciplined," "suspended," & "parents are told."
Education	"Have you ever had a class about the bad effects that smoking has on your health?"
Activities	"Do you participate in school sports?" & "Do you participate in other school activities?"
Grades & Detentions	"What type of grades do you get?" & "During this school year how many detentions and suspensions have you had?"
Subjective social status [44]	Participants were asked to indicate on a 10-rung ladder where they think their position is relative to other children attending their school.
***Neighborhood characteristics***	
Neighborhood norms	"Do you think that your neighbors would say something to you if they saw you smoking?" & "Do you think your neighbors would say something to your parents if they saw you smoking?"
Access	"If you try to buy cigarettes will you be asked to show an ID?" & "Is it easy for you to buy cigarettes in your neighborhood or near your school?"
Pro- and/or anti-smoking messages	Participants were asked whether they have seen MORE pro-smoking messages, anti-smoking messages, or neither type on billboards, television, newspapers, etc., during the previous month.

Note. All Cronbach's alphas are derived from the baseline study data.

### Variables available for analysis from the participants' parent

Because our study participants are a sub-cohort of a larger cohort (the population-based infrastructure of Mexican American households maintained by the Department of Epidemiology at UTMDACC), basic descriptive data provided by the participant's parents were available for 93.5% of the participants in the current study. These included educational attainment, nativity status (U.S. or Mexico), years living in the U.S. (assessed among participants born in Mexico only), acculturation, and smoking status.

Educational attainment was divided into three categories: less than high school, high school/General Educational Development equivalency, and more than high school. Acculturation was assessed using four items that assess linguistic proficiency in English from the Bidimensional Acculturation Scale for Hispanics (BAS), a validated acculturation instrument designed for use with Mexican American adults [13]. Scores ranged from 1 to 4, with higher scores reflecting fluency in English and higher levels of acculturation. The scale has very good internal reliability (alpha = 0.88). Smoking status was divided into two categories: ever and never. Ever smokers included current and former smokers who reported smoking at least 100 cigarettes in their lifetime.

### Statistical analyses

We conducted Student's t-tests and Pearson chi-square analyses, as appropriate, to examine the associations between smoking susceptibility (non-susceptible vs. susceptible) and children's demographics, their parent's demographics, attitudes toward smoking, peer influences, family influences, school characteristics, and neighborhood characteristics.

To identify the variables that independently predicted susceptibility, we performed an unconditional backward elimination stepwise logistic regression analysis. Prior to completing this multivariable analysis, we examined the correlations among the risk factors that demonstrated a significant bivariate association (p < 0.05) with susceptibility. Because the two peer influence variables ("three best friends smoke," "some friends smoke;" see Table 1) were correlated (r = 0.64; p < 0.01), we created a dichotomous summary measure ("any friends smoke") to use in the multivariate models. Participants who responded "none" on the "How many of your friends smoke?" question and "0" on the "How many of your closest friends smoke?" question were coded as 0; all other participants were coded as 1. However, the correlations among all other risk factors were low. Therefore all risk factors that demonstrated a significant bivariate association (p < 0.05) with susceptibility were entered into the model. All risk factors measured as scaled variables (e.g. attitudes toward smoking, family and peer norms, parental acculturation, and subjective social status) were entered as continuous variables, while the categorical variables were entered as dummy variables. To determine the extent of multicollinearity among the risk factor variables, we examined the variance inflation factor (VIF) for each variable retained in the model.

Finally, we created a cumulative risk score for each participant by summing the risk factors identified from the logistic regression. We dichotomized most risk factors assessed as continuous variables (positive outcome expectations, peer norms, temptations to try cigarettes, and subjective social status) at the median. However, we dichotomized detentions as none or at least one. We then assigned each risk factor the value of 1 and created a risk index by summing risks [14]. Cumulative risk scores ranged between 0 and 8; no participants obtained the highest possible score of 9. We then completed an unconditional logistic regression.

## Results

A total of 1,328, participants had completed the baseline cross-sectional survey. Of these, 129 participants (9.7%) were identified as ever-smokers and were excluded from the current analysis. The sample size of never-smokers available for analysis was 1,199. However, because another 12 participants had missing data on the temptations measure the sample size available for both logistic regression models was 1,187. Therefore to be consistent throughout, descriptive data tables are also based on the 1,187 participants. All analyses were conducted using SPSS.

Table 2 summarizes the demographic characteristics of the study participants and their parents by the children's smoking susceptibility. Overall, 262 participants (22.1%) were susceptible to smoking. Boys (25.6%) were more likely to be susceptible than girls (18.9%; p < 0.01). Susceptibles were on average 5 months older than non-susceptibles. There were no differences in susceptibility based on participants' country of birth, length of residency in the US, or language spoken at home, or in their parents' educational attainment. However, while not significantly different, the parents of susceptibles had lived in US for an average of 1.2 years longer than the parents of nonsuscpetibles. They were also more acculturated than the parents of the non-susceptibles (p = 0.04), and susceptible participants were more likely to have a mother who smokes or smoked than non-susceptibles (p = 0.05). The pattern was similar for fathers, but the difference was not significant, likely because of the very small number of fathers enrolled in the study.

**Table 2:** Summary statistics of children's and parents' characteristics by susceptibility status at baseline

	**N (%)**		
		
**Variable**	**Susceptible**	**Not susceptible**	**P-value**
***Children (N = 1,187)***	262 (22.1)	925 (77.9)	--
Gender			
Boys	144 (25.6)	418 (74.4)	
Girls	118 (18.9)	507 (81.1)	< 0.01
Age			
11 years	77 (15.6)	418 (84.4)	
12 years	87 (22.8)	295 (77.2)	
13 years	98 (31.6)	212 (68.4)	< 0.01
Boys, M (SD)	12.1 (0.9)	11.8 (0.8)	< 0.01
Girls, M (SD)	12.1 (0.8)	11.8 (0.8)	< 0.01
Nativity status			
Born in United States	193 (22.3)	673 (77.7)	
Born in Mexico	69 (21.5)	252 (78.5)	0.77
Years in US, M (SD)	10.53 (3.2)	10.39 (2.9)	0.52
Language spoken at home			
Spanish only spoken at home	61 (25.3)	180 (74.7)	0.22
English only spoken at home	23 (19.0)	98 (81.0)	0.35
***Parent (primary informant) (N = 1,110)***	248 (22.3)	862 (77.7)	
Educational attainment*			
Less than high school	167 (23.0)	559 (77.0)	
Completed high school	38 (19.9)	153 (80.1)	
More than high school	42 (21.8)	151 (78.2)	0.66
Nativity status*			
Born in United States	49 (25.9)	140 (74.1)	
Born in Mexico	199 (21.6)	722 (78.4)	0.20
Years in U.S., M (SD)	18.2 (11.5)	16.9 (11.7)	0.13
Acculturation*			
Low acculturation	174 (21.1)	649 (78.9)	
High acculturation	74 (25.8)	213 (74.2)	0.10
Acculturation scale, M (SD)	2.2 (0.9)	2.1 (0.9)	0.04
Self-reported smoking*			
Mother ever smoker	38 (29.0)	93 (71.0)	0.05
Father ever smoker	9 (29.0)	22 (71.0)	0.31

* Data were available for the parents if the parent was enrolled as a primary informant in the population-based infrastructure. Primary informants included 60 men and 1050 women. Parental nativity status data was missing on 1 participant; and self-reported smoking status data was missing on 18 mothers. Missing categories were not included in calculations.

M, mean; SD, standard deviation.

Table 3 presents summary statistics for the attitudinal and social risk factors studied, by susceptibility status. Four of the five attitude measures were associated with susceptibility in the expected direction. Compared to the non-susceptibles, susceptibles perceived fewer "cons" and more "pros" associated with smoking, had higher positive expectations about smoking, and reported more temptations to smoke.

**Table 3:** Attitudes toward smoking, peer influence, family influence, school characteristics, and neighborhood characteristics, by susceptibility status at baseline (N = 1,187)

	**N (%)**		
		
**Variable**	**Susceptible**	**Not susceptible**	**P-value**
***Total***	262 (22.1)	925 (77.9)	--
***Attitudes toward smoking***			
Pros of smoking^1 ^*, M (SD)	2.2 (0.7)	1.8 (0.7)	< 0.01
Cons of smoking^1 ^*, M (SD)	4.4 (1.2)	4.5 (0.7)	0.04
Temptations to try smoking^2 ^*, M (SD)	1.5 (0.6)	1.3 (0.5)	< 0.01
Positive outcome expectations^3 ^*, M (SD)	1.5 (0.5)	1.2 (0.3)	< 0.01
Negative outcome expectations^3 ^*, M (SD)	3.4 (0.6)	3.5 (0.6)	0.10
***Peer influence***			
Normative influence from peers	176 (32.4)	368 (67.6)	< 0.01
Three best friends smoke, M (SD)	0.2 (0.5)	0.1 (0.3)	0.01
Some friends smoke	59 (48.8)	62 (51.2)	< 0.01
Any friends smoke	63 (47.4)	70 (52.6)	< 0.01
***Family influence***			
Normative influence from family	74 (35.7)	133 (64.3)	< 0.01
Father currently smokes	91 (26.5)	252 (73.5)	0.02
Mother currently smokes	32 (37.6)	53 (62.4)	< 0.01
Brother currently smokes	31 (47.0)	35 (53.0)	< 0.01
Sister currently smokes	12 (44.4)	15 (55.6)	< 0.01
Other householder currently smokes	21 (24.4)	65 (75.6)	0.59
***School characteristics***			
Believes s/he can smoke on campus	50 (33.3)	100 (66.7)	< 0.01
Has taken a health class	209 (21.5)	758 (78.5)	0.28
Endorses "kids think smoking is cool"	61 (37.9)	100 (62.1)	< 0.01
Endorses "kids think smoking is stupid"	209 (20.0)	836 (80.0)	< 0.01
Participates in school sports	153 (22.1)	540 (77.9)	0.99
Participates in school extracurricular activities	157 (20.5)	610 (79.5)	0.07
Believes parents will be told if caught smoking on campus	105 (18.9)	452 (81.1)	0.02
Subjective social status*, M (SD)	7.9 (1.6)	8.4 (1.6)	< 0.01
School grades*, M (SD)	2.1 (0.8)	1.9 (0.7)	< 0.01
Detention*, M (SD)	1.3 (2.9)	0.5 (1.7)	< 0.01
***Neighborhood characteristics***			
Believes neighbors will report their smoking to their parents	201 (20.1)	799 (79.9)	< 0.01
Perceives access to buy cigarettes	15 (42.9)	20 (57.1)	< 0.01
Has seen pro messages	96 (25.1)	286 (74.9)	0.08
Has seen anti messages	247 (22.1)	873 (77.9)	0.95

* Higher scores indicate more perceived pros of smoking, more perceived cons of smoking, more temptations to try smoking, more positive expectations about smoking, more negative expectations about smoking, more of three best friends smoke, higher social status, better grades in school, and more detentions.

M, mean; SD, standard deviation.

^1 ^Decisional Balance Scale; ^2 ^Temptations to try smoking; ^3 ^Outcome expectations.

All three aspects of peer influence were associated with susceptibility in the expected direction: Susceptibles were more likely to report that their peers strongly approve of their smoking than were non-susceptibles, to report that some of their friends smoke, and to report that at least one of their three best friends smoke. Similarly, the majority of the family influence variables were also associated with susceptibility. Susceptibles were more likely than non-susceptibles to believe that their parents strongly approve of their smoking and to have a father, mother, brother, or sister who currently smokes.

Among the school characteristics, only three were not associated with susceptibility: having taken a health class in which smoking was discussed, participating in extracurricular activities, and participating in school sports. Susceptibles were more likely to believe that students could smoke on campus and to endorse the idea that "smoking is cool." Susceptibles were less likely to endorse the idea that "smoking is stupid," and to believe their parents would be told if they were caught smoking at school. Susceptibles also reported lower subjective social status at school, and more detentions but reported higher grades than non-susceptibles. Three neighborhood characteristics were also associated with susceptibility: susceptibles were less likely to believe that if neighbors saw them smoking the neighbors would report their smoking to their parents, to have seen pro-messages, and a greater proportion of susceptibles considered it easy to buy cigarettes in their neighborhood or near school.

In the stepwise multivariate logistic regression analysis, nine of the risk factors maintained statistical significance (Table 4). Specifically, reporting more positive expectations about smoking (odds ratio [OR] = 4.85), having a brother who smokes (OR = 2.65), reporting that any friends smoke (OR = 2.19), having a mother who smokes (OR = 1.92), believing that peer norms strongly support smoking (OR = 1.76), being 13 years old (OR = 1.43), reporting more temptations to smoke (OR = 1.36), reporting lower subjective social status (OR = 1.13), and having more detentions (OR = 1.08) were independent risk factors for being susceptible to smoking. The highest VIF obtained was 1.19 indicating that collinearity was not present among the nine risk factors retained in the model.

**Table 4:** Risk factors associated with susceptibility to smoking (N = 1,187)

**Characteristic**	**Odds ratio**	**95% confidence interval**
Positive outcome expectations	4.85	3.25–7.26
Brother currently smokes	2.65	1.48–4.76
Any friends smoke	2.19	1.42–3.36
Mother currently smokes	1.92	1.13–3.25
Normative influence from peers	1.76	1.27–2.43
Age 13	1.43	1.02–2.01
Temptations to try smoking	1.36	1.05–1.76
Subjective social status	1.13	1.03–1.24
Detentions	1.08	1.01–1.16

Finally, we present the results from the logistic regression model based on the cumulative risk score (Table 5). The risk for being susceptible to smoking increased with increasing number of risk factors (p_trend _< 0.01). Compared to participants with no risk factors, participants with two risk factors (23.4% of the participants) were 3.06 times more likely to be susceptible, participants with three risk factors (20.6% of the participants) were 3.88 times more likely to be susceptible to smoking, participants with four risk factors (13.1% of the participants) were 10.89 times more likely to be susceptible to smoking, and participants with five or more risk factors (14.2% of participants) were 25.67 times more likely to be susceptible to smoking.

**Table 5:** Cumulative risk associated with susceptibility to smoking (N = 1,187)

	**Overall**	
	
**No. of risk factors**	Odds Ratio*	**95% Confidence Interval**
0 (n = 131)	1.00	
1 (n = 209)	0.98	0.37–2.61
2 (n = 278)	3.06	1.34–7.03
3 (n = 245)	3.88	1.69–8.88
4 (n = 155)	10.89	4.76–24.91
5 or more (n = 169)	25.67	11.30–58.34

* P for trend < 0.01.

## Discussion

Overall, we found that 129 (9.7%) of the study participants reported that they had experimented with cigarettes. Of the remaining 1,187 never-smokers, 262 (22.1%) were susceptible to smoking. It is difficult to directly compare this percentage with those in other studies that have assessed susceptibility to smoking among Latinos because of between-study differences in the age and geographic location of the participants, as well as when and how the data were collected. For example, data collected from in-person household interviews from Mexican origin youth aged 12 to 17 in 2002 through 2004 reported a 28.8% susceptibility rate [7]. However, among a cohort of migrant Latino youth with a mean age of 13 years (standard deviation, ± 1.11), Elder *et al. *[15] reported a susceptibility rate of 35.6%, which is comparable to the rate of 31.6% we observed among the 13-year-old participants in our study (Table 2).

The principal aim of our study was to investigate the relationship between risk factors for smoking initiation and susceptibility to smoking. Most risk factors studied were associated with susceptibility to smoking, and all associations were in the expected direction. Therefore, first we focus on the results from the logistic regression models and second discuss associations that were not significant. The strongest independent risk factor for susceptibility to smoking in our study was holding positive expectations about smoking (OR = 4.85) (Table 4). Simmons-Morton *et al. *[16] too reported that youth are more likely to smoke if they think that smoking will yield socially beneficial outcomes, such as gaining more friends and gaining in popularity. Dalton *et al. *[17] found that both negative and positive outcome expectations are associated with susceptibility to smoking; however, in our study, negative expectations were not significantly associated with susceptibility to smoking. We used the outcome expectations measure developed by Dalton *et al.*, but adjusted for a wider range of variables and still found the summary measure of positive expectations to be the strongest predictor of susceptibility to smoking. Our results, therefore, are consistent with previous findings [16,17] and combined with the previous studies' results underscore the importance of variables, such as outcome expectations, as predictors of smoking initiation.

In general there is a robust association among sibling's smoking status [18]; in particular smoking by older siblings predicts smoking among younger siblings [19]. We found that having an older brother who currently smokes is associated with a two fold increased odds of being susceptibility to smoking (OR = 2.65). Recent studies demonstrated that having a parent who smokes or smoked is another strong and consistent predictor of smoking initiation [20-26], while parental expectations not to smoke are protective [27]. We examined the role of the fathers' smoking status independent from that of the mothers' smoking status. Although a higher percentage of fathers than mothers were current smokers, only the mothers' smoking status was associated with their children's susceptibility (OR = 1.92). *Familismo*, which refers to the belief that the needs of the family outweigh the needs of the individual, plays a central role in how Mexican and Mexican American families operate. One component of *familismo *is the obligation to provide material and emotional support to family members [28]. In many families, this is realized by the father working long hours while the mother stays at home to raise the children and take care of the home. As a result, children spend many more hours with their mothers than fathers and form a stronger emotional bond with their mothers. It is possible, therefore, that the mothers' behaviors, including smoking behaviors, exert a stronger influence on children than the fathers' behaviors. The findings that both mother and brother smoking are associated with susceptibility underscores the need to focus on the family context when developing primary prevention messages targeted to Mexican origin youth.

The roles of both peer smoking and perceived peer norms have been examined extensively in studies of adolescent smoking. Research has typically found a strong association between participants' smoking status and close friends' smoking status [29-31]. We also found that friends' smoking was a risk factor for susceptibility to smoking (OR = 2.19). Because people tend to choose their friends based on shared characteristics [32], one of which could be smoking status, having close friends who smoke does not mean that it was the friends who caused the participant to smoke. However, one aspect of the susceptibility to smoking construct is lacking a firm commitment not to smoke if offered a cigarette by a friend. In addition, our finding that peer approval of smoking is a risk factor for susceptibility to smoking (OR = 1.76) is consistent with previous studies [33,34]. Together, these results suggest that the peer social context in which youths find themselves plays an important role in determining susceptibility to smoking and warrants further research in this population.

Previous research has demonstrated that older adolescents are significantly more likely to be susceptible to smoking [2,15], have experimented with cigarettes [35], and smoke than their younger peers [36]. Our findings are consistent with this well-established risk factor.

Temptations to smoke are typically examined among current smokers and recent quitters [37,38], though temptations to try smoking have been examined as predictors of smoking initiation [39]. To the best of our knowledge, no studies have examined the relationship between temptations to try cigarettes and susceptibility to smoking. The temptations measure assesses how tempted the participant is to try cigarettes in positive social situations, as a means of coping with negative affect, and to satisfy curiosity. We found that participants who reported more temptations to try cigarettes had a higher risk of susceptibility to smoking (OR = 1.36). This finding is consistent with previous studies that reported curiosity [40] and socializing with peers [41,42] as two of the major reasons why adolescents start smoking.

To date, one study has examined the relationship between subjective social status and smoking, and none have used susceptibility to smoking as an outcome variable. Finkelstein *et al. *[43] found that among adolescents in grades 7 through 12, those with lower social status were at increased risk of smoking at baseline and initiating smoking during the subsequent year. Using the same measure of subjective social status [44], similarly we found that participants who reported lower perceived social status were at greater risk for being susceptible to smoking (OR = 1.13).

Finally, we found that having had one or more detentions at school was significantly associated with being susceptible to smoking (OR = 1.08). Our finding is consistent with previous research that demonstrates detentions are associated with being susceptible [2,45] and smoking [46].

Some factors were not associated with susceptibility. Neither the child's country of birth nor the language spoken at home was related to susceptibility status. This is consistent with the finding of Gritz *et al. *[2] that level of cultural identification was not associated with smoking susceptibility among Latino youth. Parental educational attainment also was not related to children's susceptibility status, although previous studies have noted an inverse relationship between parental educational attainment and smoking [47,48].

The majority of participants (81%) reported having taken a class in school in which the bad effects of smoking on health were discussed; however, this variable did not impact susceptibility. Evaluations of school-based interventions designed to prevent smoking have demonstrated that knowledge-based interventions alone do not impact behavior [49], while those that teach resistance skills do [50]. In the current study, we do not know what content was presented and discussed in the classes or whether resistance skills were taught.

Studies investigating the association between smoking and participation in school sports have yielded mixed results. While most have found participation in school sports to be protective against smoking [51-53], others have found school sports to be associated with higher rates of smoking [54]. We found no association. It is possible that we did not observe an association because according to local school district policy, only students in the 7^th ^grade and above participate in school sports. Most of our study participants were in the 5^th ^or 6^th ^grade when they enrolled in the study, and those who did answer the question about participation in school sports were likely answering about their participation in physical education classes rather than school sports.

Previous research has demonstrated that messages perceived as pro-tobacco and those perceived as anti-tobacco influence susceptibility to smoking [55] and that exposure to pro-tobacco media and advertising does increase susceptibility to smoking over time [56]. In the current study, these associations were not statistically significant.

Our study has both strengths and limitations. A strength of our study pertains to the large sample size, which allowed us to ascertain the number of co-occurring risk factors that tip the balance from non-susceptible to susceptible. This analysis, based on a cumulative risk score, revealed no differences in susceptibility among participants with zero or one risk factors. However, among the 72% of participants with two or more risk factors, the chances of being susceptible to smoking increased with the number of risk factors. Indeed, it was striking that the 169 participants (14.2% of participants) with five or more risk factors were over 20 times more likely to be susceptible compared to their peers with no risk factors.

Our study also generated some novel findings. To our knowledge, our study is the first to report that low subjective social status and temptations to smoke are associated with susceptibility to smoking. Future research will need to confirm these findings and determine if either or both risk factors generalize to other populations. Other strengths of the study include the fact that our participants were from a population-based cohort and included roughly equal numbers of girls and boys. In addition, we used validated measures and collected the data in the participants' homes using PDAs to ensure their privacy. A final strength of the study was our ability to recruit a large sample of Mexican origin participants, which is an understudied population.

One limitation of this study was that we were not able to examine the relationship between depression and susceptibility to smoking. We also did not have biochemical validation of the participants' smoking status (e.g., cotinine levels in saliva). However, we informed participants during the consent process that they might be selected to provide a saliva sample to check their smoking status; this "bogus pipeline" procedure has been shown to increase the validity of self-reported smoking status [57].

## Conclusion

In summary, compared to non-susceptible participants, smoking-susceptible participants were more likely to hold more positive expectations about smoking, have a brother who smoked, report that their friends smoked, have a mother who smoked, believe their peers approve of their smoking, be older, report more temptations to try smoking, report lower subjective social status at school, and have had a detention during the school year. Overall, the strongest risk factor we identified was holding positive expectations about smoking, although both family- and school-based characteristics were important, too. These findings can be used to inform the development of culturally sensitive primary prevention programs.

School-based interventions that target positive expectations about smoking, the role that peers may play in promoting positive expectations, and potential differences in positive expectations among students with differing levels of social status, may be useful in this population. In addition, the findings that having a mother and a brother who smoke increase the risk of being susceptibility underscore the continued need to develop family- and community-based primary prevention programs.

## Competing interests

The authors declare that they have no competing interests.

## Authors' contributions

AVW and AJW took the leads in writing the manuscript, computed initial analysis, and interpreted the data. VV completed additional analysis, coordinated the drafting of the manuscript, and was responsible for the referencing. MLB and AVP interpreted the results and provided critical feedback on the drafts. MRS conceived the study, interpreted the results, and provided critical revisions.

## Pre-publication history

The pre-publication history for this paper can be accessed here:


